# Identification of Genes Required for Secretion of the *Francisella* Oxidative Burst-Inhibiting Acid Phosphatase AcpA

**DOI:** 10.3389/fmicb.2016.00605

**Published:** 2016-04-28

**Authors:** Ky Van Hoang, Carolyn G. Chen, Jacob Koopman, Jasmine Moshiri, Haley E. Adcox, John S. Gunn

**Affiliations:** Center for Microbial Interface Biology, Department of Microbial Infection and Immunity, The Ohio State UniversityColumbus, OH, USA

**Keywords:** *Francisella*, protein secretion, acid phosphatase

## Abstract

*Francisella tularensis* is a Tier 1 bioterror threat and the intracellular pathogen responsible for tularemia in humans and animals. Upon entry into the host, *Francisella* uses multiple mechanisms to evade killing. Our previous studies have shown that after entering its primary cellular host, the macrophage, *Francisella* immediately suppresses the oxidative burst by secreting a series of acid phosphatases including AcpA-B-C and HapA, thereby evading the innate immune response of the macrophage and enhancing survival and further infection. However, the mechanism of acid phosphatase secretion by *Francisella* is still unknown. In this study, we screened for genes required for AcpA secretion in *Francisella*. We initially demonstrated that the known secretion systems, the putative *Francisella*-pathogenicity island (FPI)-encoded Type VI secretion system and the Type IV pili, do not secrete AcpA. Using random transposon mutagenesis in conjunction with ELISA, Western blotting and acid phosphatase enzymatic assays, a transposon library of 5450 mutants was screened for strains with a minimum 1.5-fold decrease in secreted (culture supernatant) AcpA, but no defect in cytosolic AcpA. Three mutants with decreased supernatant AcpA were identified. The transposon insertion sites of these mutants were revealed by direct genomic sequencing or inverse-PCR and sequencing. One of these mutants has a severe defect in AcpA secretion (at least 85% decrease) and is a predicted hypothetical inner membrane protein. Interestingly, this mutant also affected the secretion of the FPI-encoded protein, VgrG. Thus, this screen identified novel protein secretion factors involved in the subversion of host defenses.

## Introduction

*Francisella tularensis*, a gram-negative facultative intracellular pathogen and the causative agent of the life-threatening disease tularemia in humans and other zoonotic animals, is one of the most infectious bacterial pathogens known (Oyston et al., 2004). Pneumonic infection with less than 10 colony-forming units of the type A strain can lead to a fatal infection if left untreated (Oyston, 2008). Other routes of infection include ingestion of the contaminated water and food, exposure to infected animals or transmission from an arthropod vectors. In the infected host, *Francisella* is able to infect many cell types including mononuclear phagocytes, epithelial cells and hepatocytes; however, the primary target of *F. tularensis* is the macrophage. *Francisella* primarily enters the macrophage by phagocytosis wherein the bacteria block the fusion of *Francisella*-containing phagosomes with lysosomes and later escape into the cytosol where it rapidly proliferates to high numbers, subsequently inducing cell death (Clemens et al., 2004; Mohapatra et al., 2007; Santic et al., 2008).

*Francisella* uses multiple strategies to counteract the host innate defense mechanisms (Jones et al., 2012) including expression of the Type IV pili and Type VI secretion systems (Hager et al., 2006), modification of lipooligosaccharides (Gunn and Ernst, 2007; Soni et al., 2010) on the bacterial outer membrane, and the utilization of complement receptor (CR3) to subvert the immune response (Dai et al., 2013). Our previous studies have shown that *Francisella* has at least four acid phosphatases Acp (-A,-B,-C, and HapA) that suppress the oxidative burst after the bacterium is phagocytosed into the host cells (Mohapatra et al., 2010). The effects of these acid phosphatases allow *Francisella* to elicit disease and evade intracellular killing by macrophages (Mohapatra et al., 2007), as acid phosphatase deletion mutants were more susceptible to intracellular killing and delayed in escaping from macrophage phagosomes (Mohapatra et al., 2007). The most active acid phosphatase, AcpA, was demonstrated to be secreted both *in vitro* and inside infected macrophages, and altered phosphorylation of p47(phox) and p40(phox) (Mohapatra et al., 2010). However, the mechanism of acid phosphatase secretion in *Francisella* is still unknown.

One of the major virulent strategies utilized by Gram-negative bacterial pathogens to subvert host defenses is the assembly of the specialized secretion machinery within the bacterial envelope. At least six secretion systems (Types I-VI) in Gram-negative bacteria have been described (Desvaux et al., 2009). These secretion machineries enable bacteria to deliver proteins, DNA, and small molecules into the environment or into recipient cells. There have been limited studies on secretion systems in *Francisella.* Comparative genomic analysis of *Francisella* species revealed that *Francisella* do not encode for functional Types III and IV secretion systems in their genomes (Champion et al., 2009). It has been demonstrated that the elements of the Sec secretion system contribute to biofilm formation in *Francisella*, however, whether this system is involved in extracellular protein secretion is still unknown (Margolis et al., 2010). To the best of our knowledge, in addition to *Francisella* outer membrane vesicles (McCaig et al., 2013), Type IV pili and the *Francisella* pathogenicity island (FPI)-encoded Type VI secretion system are the only known systems involved in *Francisella* extracellular protein secretion (Maier et al., 2004; Hager et al., 2006; Hare and Hueffer, 2014). In this study, we demonstrated that Type IV pili and the FPI-encoded Type VI secretion system are not responsible for AcpA secretion. We developed the experiment approaches for the optimal detection of AcpA in the culture supernatant and subsequently screened for transposon mutants deficient in AcpA secretion, leading to the identification of new loci involved in protein export in *Francisella*.

## Materials and Methods

### Bacterial Growth Conditions

The wild-type (WT) *F. novicida* U112 strain with or without the *acpA* expression plasmid (p*acpA*-Flag) was used in this study. When needed, *F. novicida* transposon mutants were obtained from the BEI Resources comprehensive *F. novicida* mutant library^1^. Bacteria were grown at 37°C in modified tryptic soy broth (mTSB) or agar by supplementing with 135 μg/ml ferric pyrophosphate and 0.1% cysteine hydrochloride. When appropriate, the growth medium and agar were supplemented with tetracycline (12 μg/ml) and/or kanamycin (17 μg/ml). *Escherichia coli* DH5αaaa*pir* strain containing the pFNLTP16 Himar1 transposon plasmid as used previously in Maier et al. (2006) was grown on Luria-Bertani (LB) agar containing kanamycin (45 μg/ml) at 37°C. The transposon-containing plasmid was purified from *E. coli* DH5αλ*pir* cells as described (Maier et al., 2006).

### Establishment of a Screen for Mutants with Decreased AcpA in the Culture Supernatant

An ultracentrifugation scheme described in our previous study (Dai et al., 2012) (10,000 × *g* for 20 min at 4°C to remove cell pellets and 150,000 × *g* for 135 min 4°C to remove cell debris and membrane vesicles) can be used to separate cells, bacterial membrane vesicles, and cell debris from protein-containing bacterial supernatants. However, this method is only feasible with small numbers of samples. To detect secreted AcpA in the culture supernatant in our screen, different centrifugation strategies were examined to optimally remove cells and cell debris from the bacterial culture supernatants. Both WT bacteria and the WT strain carrying the *acpA* expression plasmid (p*acpA*-Flag) were tested. The conditions included three different centrifugation schemes: (1) 16,100 × *g* once for 30 min in 1.5 ml microfuge tubes, (2) in a 96-well format, 2095 × *g* once for 40 min, and (3) in a 96-well format, 2095 × *g* for 40 min with the transfer of supernatants to another plate and centrifugation at 2095 × *g* for an additional 40 min. The ultracentrifugation scheme that was described in our previous study was used as a control (Dai et al., 2012). A volume of 50 μl of supernatant was dried in a 96-well immune-plate (Thermo Scientific, Rochester, NY, USA) overnight in the presence of protease inhibitor and then used for AcpA ELISA analysis as described below. The bacterial membrane protein FopA was included as a negative control. The centrifugation scheme that optimally excluded FopA from the supernatant was chosen for the screen. Following this, we also varied pH (6, 7, 8, and 9) and time (7, 12, and 22 h) after culture inoculation for the optimal secretion of AcpA, monitoring by ELISA.

### Random Transposon Mutagenesis

The temperature-sensitive transposon plasmid pFNLTP16 Himar1 H3 containing a kanamycin resistance cassette was used as described previously (Maier et al., 2006). Briefly, *F. novicida* containing p*acpA*-Flag was grown on mTBS agar containing 12 μg/ml of tetracycline overnight, collected from plates, and washed four times with 0.5 M sucrose. pFNLTP16 was introduced into the bacteria through electroporation (Maier et al., 2006). Electroporated cells were then non-selectively incubated while shaking at 150 rpm at 30°C. After 4 h of incubation, the cells were plated on mTSB agar plates supplemented with 17 μg kanamycin/ml and 12 μg tetracycline/ml. After 48 h of incubation at 40°C (conditions eliminate the temperature-sensitive plasmid and are favorable for the integration of the kanamycin resistant cassette into bacterial chromosome), a transposon library of the mutants was compiled by picking colonies from mTSB agar plates and inoculated into mTSB plus tetracycline (12 μg/ml) and kanamycin (17 μg/ml) in a 96-well plate.

### Screening Mutants with Increased/Decreased AcpA Secretion

To screen transposon mutants for increased/decreased AcpA secretion, the transposon mutant library was replicated by transferring 5 μl of the overnight culture from 96-well plate format into new 96-well plates containing 240 μl mTSB pH 7.5 plus tetracycline (12 μg/ml) and kanamycin (17 μg/ml) and incubated at 37°C. After 22 h incubation (OD_600_ = 0.3), 96-well plates were centrifuged at 2095 × *g* at 4°C for 40 min. and then 150 μl of the supernatant from each well was then transferred onto a new 96-well plate, which was centrifuged at 2095 × *g* at 4°C for additional 40 min. 50 μl of the supernatant from each well was then transferred to an immune-plate (Thermo Scientific, NY) containing 10 μl protease inhibitor solution (1 tablet in 1 ml phosphate buffered saline (PBS) (Complete Mini, Roche, IN, USA) and dried overnight. AcpA levels were analyzed by ELISA. Briefly, the plates were washed three times with PBS and blocked with 1% bovine serum albumin (BSA) in PBS (blocking buffer) for 2 h at room temperature. The plate was then incubated with rabbit anti-AcpA antibodies (1:1000) in blocking buffer. After 2 h at room temperature, the plate was washed three times with PBS and subjected to secondary horse radish peroxidase conjugated goat anti-rabbit antibodies (BioRad, Hercules, CA, USA) (1:1000) in blocking buffer for 1 h at room temperature. Colorimetric detection was performed with the ECL kit reagents by the manufacturer’s protocol (BioRad, Hercules, CA, USA) and the plate was read by a plate reader at OD_415_. Mutants without a growth defect and with more than a 1.5-fold decrease or increase in OD_415_ were chosen for a second screening.

### Determination of Insertion Sites in Transposon Mutants

Genomic DNA of potential mutants from the second screening was purified as described previously (Maier et al., 2006). The transposon insertion sites were determined by direct sequencing using the transposon primer Kan-2 FP-1 (Gallagher et al., 2007) (5′-ACCTACAACAAACTCTCATCAACC-3′). For those mutants whose transposon insertion sites were unable to be identified by direct sequencing, inverse-PCR was applied. Briefly, genomic DNA (gDNA) of each mutant was purified and digested with NlaIII which does not cut within the transposon. The digested gDNA was used as a template for PCR amplification using inverse PCR primers (forward primer 5′-TCAATTCGAGCTCGGGTATC-3′ and reverse primer 5′-ACCGTAAAGCACGAGGAAGC-3′). The PCR products were separated on an agarose gel, purified and subjected to sequencing using the forward primer. The obtained sequences were aligned to the *F. novicida* genome to identify the transposon insertion sites.

### Western Blot Analysis

Overnight cultures (0.5 ml) of candidate mutants with or without the *acpA* expression plasmid p*acpA*-Flag were inoculated into 45 ml mTSB and grown until an optical density (OD_600_) of 0.3. The cultures were then subjected to ultracentrifugation to remove cells and cell debris from supernatants. The proteins from the cell lysates and the supernatants were collected as described previously (Dai et al., 2012). In brief, the cell pellets collected by ultracentrifugation 10,000 × *g* for 20 min at 4°C were lysed by sonication and cell lysates from each strain were obtained. The supernatants were filtered and ultra-centrifuged at 150,000 × *g* for 135 min at 4°C to remove cell debris and membrane vesicles. Trichloroacetic acid (TCA) was added (10% v/v) and kept at 4°C overnight. The precipitated proteins were collected by ultracentrifugation at 70,000 × *g* for 1 h. Pellets were washed with acetone, dried at room temperature and re-suspended in PBS. Protein concentration of the cell lysate and supernatant was determined by a bicinchoninic acid (BCA) assay (Thermo Fisher Scientific Inc., Waltham, MA, USA). Equal amounts of protein from the cell lysates and the supernatants from each strain were mixed with 2x SDS-PAGE sample buffer followed by SDS-PAGE and then Western blotting analysis with a mouse monoclonal anti-FLAG antibody (Sigma–Aldrich, St. Louis, MO, USA) or a rabbit polyclonal antibody against *F. novicida* AcpA (Dai et al., 2012). The ECL signal was quantified using a scanner and a densitometry program (ImageJ). To quantify the secreted proteins in the supernatant, we subtracted the background, normalized the signal to the lysate, and plotted the values as percent increase over the WT sample.

### Acid Phosphatase Activity Assays

Acid phosphatase activity of the identified mutants was measured as described in our previous publication (Mohapatra et al., 2013) with minor modification. Briefly, 0.5 ml of overnight culture of each bacterial strain was inoculated in 45 ml mTSB pH 7.5 in a 125 ml conical flask. The flasks were incubated at 37°C with shaking at 150 rpm. The bacterial cell pellets were collected at early log phase (OD_600_ = 0.3) by centrifuging at 10,000 × *g* for 20 min at 4°C. The cell pellets were washed with phosphate buffer saline (PBS) and subjected to sonication. The cell lysate was centrifuged at 16,000 × *g* for 10 min and the total protein in whole cell lysate was determined using the BCA protein assay kit. Equivalent amounts of the culture supernatant were then centrifuged at 150,000 × *g* for 135 min at 4°C using ultracentrifugation to remove cell debris and membrane vesicles. Forty microgram of the total protein from whole cell lysate in 50 μl PBS and 50 μl original supernatant from each mutant was used to examine acid phosphatase activity in the cell lysate and the culture supernatant, respectively. The acid phosphatase activity was measured using 6,8-difluoro-4-methylumbelliferyl phosphate (DiFMUP, Invitrogen) as a substrate. Fifty microliter of the 200 μM DiFMUP working solution was added into 50 μl of sample or control (PBS for cell lysate and mTSB for supernatant) in a 96-black well plate (Fisher Healthcare, Houston, TX, USA) and the reaction was incubated at room temperature for 30 min. The mean fluorescence units of the samples and the control were determined by a plate reader (excitation at ∼360 nm and emission detection at ∼460 nm). The mutants with no difference in acid phosphatase activity in the whole cell lysate but with significantly decreased activity in the supernatant compared with the WT strain were considered to have a defect in acid phosphatase secretion.

### Intramacrophage Survival Assays

The growth of bacterial strains in J774.1 murine macrophages were performed as previously described (Mohapatra et al., 2007) with minor modification. Briefly, J774.1 cells were seeded and incubated overnight with approximately 2.5 × 10^5^ cells/well. 1.25 × 10^7^ bacteria were added in each well [multiplicity of infection (MOI) of 50:1]. The plates were centrifuged at 233 × *g* for 5 min to synchronize the infection. After 2 h of incubation at 37°C and 5% CO_2_, gentamicin was added to the final concentration of 50 μg/ml to eliminate the extracellular bacteria. After 30 min, the infected cells were washed three times with pre-warmed DMEM supplemented with 10% FBS. The cells were then maintained in 10% FBS in DMEM with 10 μg gentamicin/ml to inhibit extracellular bacterial growth. At the indicated time-points, the infected cells were lysed with 0.1% Triton-X in DMEM and the total bacteria were enumerated by serial dilution and plating on mTSB agar plates.

### Statistical and Structural Analysis

Data are presented as mean ± standard deviation (SD). *p*-values were calculated using one-way ANOVA for multiple comparisons and adjusted with Bonferroni’s correction ^∗^*p* < 0.05; ^∗∗^*p* < 0.01; ^∗∗∗^*p* < 0.001; NS, not significant. Statistical analysis was performed using GraphPad Prism 5. Structural protein predictions were performed a using the I-TASSER Suite (Yang et al., 2015).

## Results

### Type IV Pili Do Not Secrete AcpA

**FIGURE 1:**
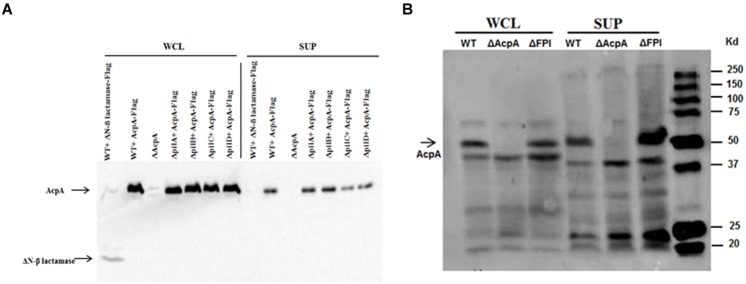
**Type IV pili and FPI are not mediating AcpA secretion in *F. novicida.* (A)** *pilA*, *pilB*, and *pilC* mutants together with wild-type (WT) *F. novicida* containing an *acpA*-Flag expression plasmid were grown in mTSB pH 7.5 to an OD_600_ of 0.3. The cells and cell debris were removed from cultures by ultracentrifugation at 10,000 × *g* for 20 min and 150,000 × *g* for 135 min. Equal amounts of total protein from the whole cell lysates and the supernatants were separated on SDS-PAGE followed by Western blotting using mouse anti-Flag antibodies. AcpA was detected in the supernatants of *pilA*, *pilB*, and *pilC* mutants. As a control, a non-secreted protein (N-terminal deletion of β-lactamase) was found in the whole cell lysate but not in the supernatants, indicating that the ultracentrifugation removed cell debris and membrane vesicles. **(B)** The *F. novicida* FPI encoding the Type VI secretion system is not responsible for the secretion of AcpA. WT *F. novicida* and FPI mutant strain were prepared as in **(A)**. Equal amounts of total protein from the whole cell lysates and the supernatants were separated on SDS-PAGE followed by Western blotting using mouse anti-Flag antibodies. AcpA was detected in the supernatants of the WT and the FPI mutant.

Type IV pili are flexible filamentous appendages on the surface of many Gram negative bacterial pathogens that have been associated with specific virulence phenotypes including adhesion, twitching motility, biofilm formation, and secretion of proteases and colonization factors (Craig and Li, 2008). *F. novicida* Type IV pili have been shown to secrete several proteins including chitinases (A and B), β-glucosidase (BglX), and a protease (PepO) (Hager et al., 2006). To examine whether this pilus mediates AcpA secretion, a *acpA*-Flag expression plasmid was introduced into *pilA*, *pilB*, *pilC*, and *pilD* mutants that were obtained from a comprehensive mutant library (Gallagher et al., 2007). A ΔN-β galactosidase-Flag expression plasmid was included as a negative control for secretion into the supernatant since ΔN-β galactosidase can not be secreted (Barker et al., 2009; Dai et al., 2012). These *pil* mutants and WT *F. novicida* all containing p*acpA*-Flag were cultured in mTBS until early log phase. The cells and cell debris were separated from cultures by ultracentrifugation. As shown in **Figure 1A**, AcpA and ΔN-β galactosidase were present in the whole cell lysates of all strains examined while AcpA, but not the non-secreted ΔN-β galactosidase control, were detected in the supernatants of all *pil* mutants (even upon longer exposure of the blots). This provided strong evidence that Type IV pili are not responsible for ApcA secretion in *F. novicida*.

### The *F. novicida* Pathogenicity Island (FPI)-Encoded Type VI Secretion System Is Not Involved in AcpA Secretion

The FPI is a cluster of 17 genes required for intracellular growth, escape into the cytosol of macrophages and virulence in a mouse model of tularemia (Maier et al., 2004; Barker et al., 2009). Although some typical components of a Type VI secretion system are missing, it has been reported that the FPI encodes for a functional Type VI secretion system in *Francisella* (Barker et al., 2009). In fact, the FPI is involved in the secretion of several proteins within the FPI including PdpA and PdpE (Barker et al., 2009; Broms et al., 2012; Hare and Hueffer, 2014). To identify whether AcpA is a potential substrate for the FPI, we compared AcpA in the supernatant of WT *F. novicida* to that of an isogenic FPI deletion mutant. The strains were grown in mTSB until the early log phase and the secreted proteins were collected using ultracentrifugation. Whole cell lysates and supernatant fractions were separated on SDS-PAGE following by Western blotting using rabbit anti-AcpA antibodies. As shown in **Figure 1B**, AcpA was detected in the supernatant of the FPI mutant, suggesting that the FPI is not responsible for the secretion of AcpA in *F. novicida*. An *acpA* mutant was included as a negative control for which AcpA was not detected in either the whole cell lysate or the supernatant.

### Optimization of Conditions to Rapidly Detect AcpA Secretion

In order to effectively screen for transposon mutants with either noticeably decreased or increased AcpA secretion via ELISA analysis, we needed a rapid, 96-well technique to reliably detect AcpA in the supernatant. Various centrifugation strategies were applied to separate intracellular and membrane-associated AcpA from secreted AcpA, and FopA was used as a membrane protein control (Supplementary Figure S1). Comparing various centrifugation methods to a ultracentrifuge isolation scheme that has been demonstrated to completely separate cells and cell debris from culture (10,000 × *g* for 20 min at 4°C to remove cell pellets and 150,000 × *g* for 135 min 4°C), we found that centrifuging twice at 2095 × *g* at 4°C for 40 min each optimally excluded the membrane protein FopA from the supernatant (Dai et al., 2012). Other centrifugation schemes did not optimally separate cells and cell debris from the bacterial cultures.

**FIGURE 2:**
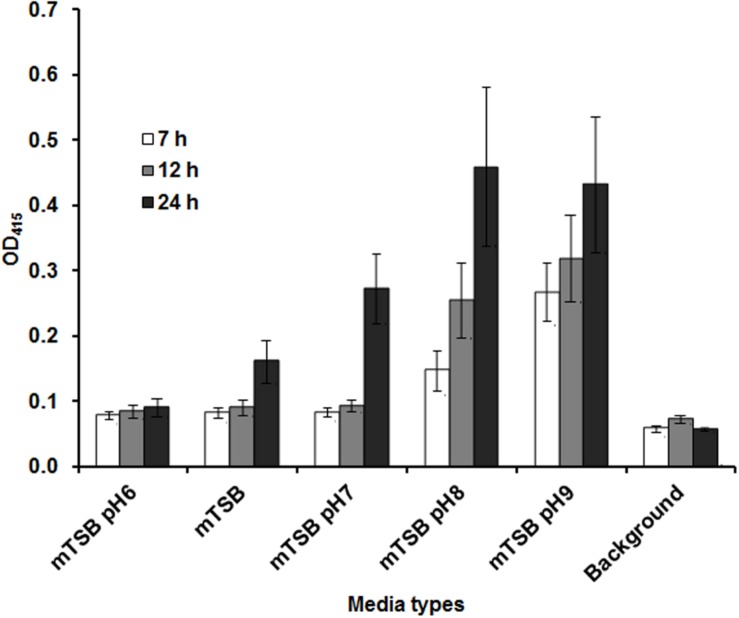
**Amount of AcpA in the culture supernatant is dependent on the pH of the medium and the growth phase.** Wild-type (WT) *F. novicida* carrying the *acpA*-Flag expression plasmid was cultured in 240 μl mTSB with different pH conditions (pH 6, 7, 8, and 9) statically in 96-well plates. At different time-points (7, 12, and 22 h), the plates were centrifuged at 2095 × *g* for 40 min and 150 μl supernatant from each well was transferred onto a new 96-well plate and centrifuged for an additional 40 min at 2095 × *g.* Fifty microliters of the supernatant from each well was subjected to an AcpA ELISA. mTSB was included as a background control (with respect to 22 h time-point). The data were presented as a representative of three independent experiments.

Our previous studies indicated that *Francisella* AcpA is secreted in relatively low amounts into the culture supernatants in regular media (mTSB), thus it would be difficult to detect a difference in AcpA secretion between mutants and the WT strain. We reasoned that AcpA secretion may be regulated by environmental factors such as pH, temperature, and growth phase; therefore, *F. novicida* containing plasmid-borne AcpA was cultured in mTSB of differing pH conditions (pH = 6, 7, 8, and 9) and monitored at various stages of the growth. Analysis of the supernatants indicated that detection of AcpA was growth phase and pH dependent with maximal levels of the secreted AcpA observed in mTSB of pH 8 at 22 h post inoculation (OD_600_ of 0.3) (**Figure 2**). However, this pH condition resulted in a slightly slower growth of *Francisella* and thus mTSB pH of 7.5, which did not show a similar growth deficiency, was used for the optimal detection of the secreted AcpA.

### Transposon Mutagenesis and Screening for Mutants with Decreased AcpA Secretion

The workflow of the identification of genes involved in AcpA secretion is described in Supplementary Figure S2. A transposon mutant library was constructed by the introduction of plasmid pFNLTP16 Himar1 H3 into *F. novicida* containing p*acpA-*Flag. A total of 5,450 mutants were screened by ELISA for altered levels of AcpA in the supernatants using the growth conditions and the optimized detection technique described above. While not a saturating screen, it identified 53 mutants with decreased AcpA levels and 4 mutants with an increased AcpA levels (at least twofold compared to WT). A secondary screening using an AcpA ELISA detection method of these 53 mutants resulted in a reduction to 24 candidate mutants with decreased AcpA secretion and no mutants with increased AcpA in the supernatant. The genomic DNA of these 24 mutants was purified and sequenced using the transposon specific primer Kan-2 FP-1 (Gallagher et al., 2007). Sequencing of inverse-PCR products was applied for the mutants in which the identity of the transposon location was unable to be determined by direct sequencing. These sequences were then searched against the *F. novicida* genome and the transposon insertion site for each candidate mutant was determined.

**FIGURE 3:**
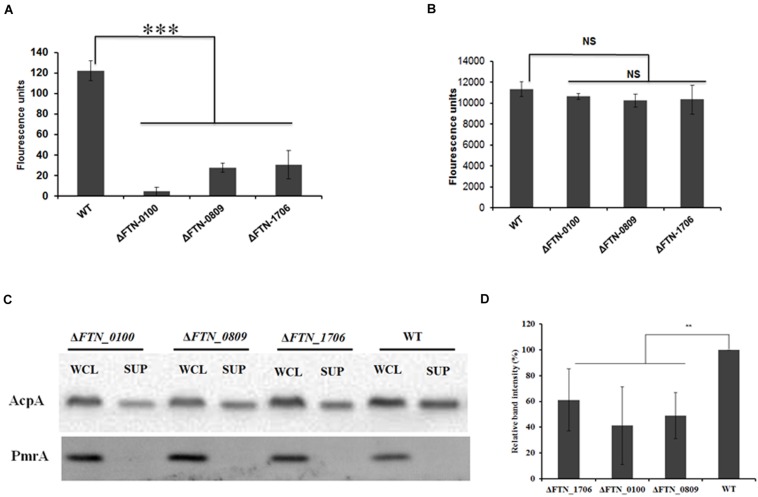
**Confirmation of genes involved in AcpA secretion.** Δ*FTN_0100*, Δ*FTN_0809*, and Δ*FTN_1706* mutants carrying the *acpA*-Flag expression plasmid were cultured in mTSB pH 7.5 in 125ml conical flashes until an OD_600_ of 0.3. Proteins from supernatants were collected by ultracentrifugation and TCA precipitation. Proteins from cell lysates were obtained by sonication. Equal amounts of proteins from the supernatants **(A)** and the cell lysates **(B)** were used for acid phosphatase enzymatic assays. In parallel, Western blotting was performed to compare AcpA levels between mutants and the WT strain using an anti-Flag antibody. A PmrA Western blot was included as negative control for supernatant purity **(C)**. Densitometry data for AcpA Western blots were evaluated from three independent experiments **(D)**. (^∗∗^*P* < 0.01, ^∗∗∗^*P* < 0.001, NS, not significant, one way-ANOVA).

To examine if the genes identified in our transposon library were indeed involved in the secretion of AcpA, an independent evaluation of these loci was performed. An AcpA-Flag-encoding plasmid was introduced into the corresponding mutants obtained from the BEI Resources *F. novicida* transposon library. AcpA from supernatants and cell lysates of these mutants was compared to the WT strain by Western blotting using the AcpA antibody as well as by acid phosphatase activity. Three mutants with a decrease in acid phosphatase activity (**Figures 3A,B**) and AcpA protein (**Figures 3C,D**) in the supernatants but not in the cell lysates were confirmed (**Figure 3**). Two mutants with moderately (60%) decreased acid phosphatase activity in supernatants (FTN_0809; transposon insertion site at 433/1251 bp and FTN_1706; transposon insertion site at 1074/1182 bp) were annotated as transporters belonging to major facilitator superfamily (MFS). The other mutant (FTN_0100; transposon insertion site at 219/1017 bp) demonstrated a dramatic decrease (85%) of supernatant acid phosphatase activity and was annotated as a hypothetical protein. FTN_0100 does not appear to be part of an operon and there is no downstream gene transcribed from the same strand of the chromosome. This gene is predicted to be composed of nine helical transmembrane domains (Supplemental Figure S3) and while found in *F*. *tularensis* SCHUS4 and *F. tularensis holarctica*, it contains an N-terminal truncation in these subspecies.

### The FTN_0100 Mutant Is Defective in Intramacrophage Survival

**FIGURE 4:**
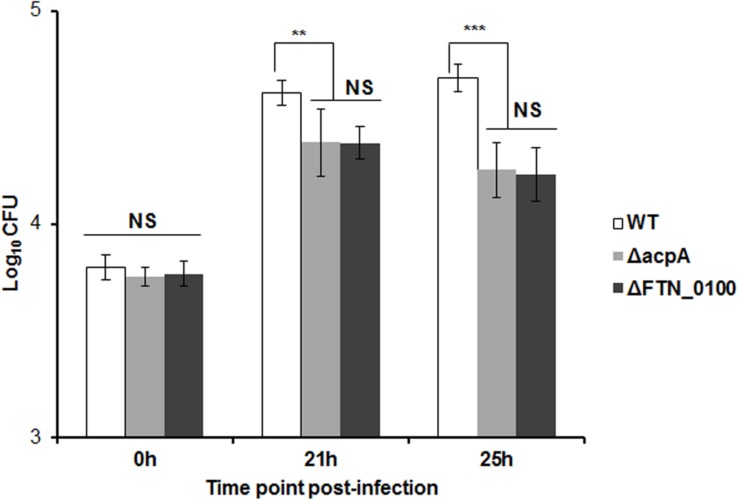
**Intracellular growth of the *FTN_0100* mutant.** The growth of the *FTN_0100* mutant in J774.1 murine macrophages was examined. As controls, WT and *acpA* mutant strains were included. The data presented as a representative of two independent experiments (^∗∗^*P* < 0.01, ^∗∗∗^*P* < 0.001, NS, not significant, One-way ANOVA analysis).

In the infected host, *Francisella* primarily targets the macrophage (Fortier et al., 1992; Jones et al., 2012). In our previous study, the AcpA mutant was defective in intracellular growth in macrophages (Mohapatra et al., 2007). To determine if reduced AcpA secretion in the *FTN_0100* mutant resulted in a similar phenotype, we evaluated the survival of this strain in J774.1 murine macrophages. As shown in **Figure 4**, there was no difference in the initial bacterial phagocytosis among the tested strains. While all strains replicated in J774.1cells, the *FTN_0100* mutant showed about a half-log reduction at 12 and 24 hrs post-infection, which was nearly identical to the survival of the *acpA* mutant. These results suggest that the reduced secretion of AcpA in the *FTN_0100* mutant has an impact on intracellular survival and indicates an important role of FTN_0100 in *Francisella* pathogenesis.

### The FTN_0100 Mutant Affects Secretion of the FPI Protein Component VgrG

**FIGURE 5:**
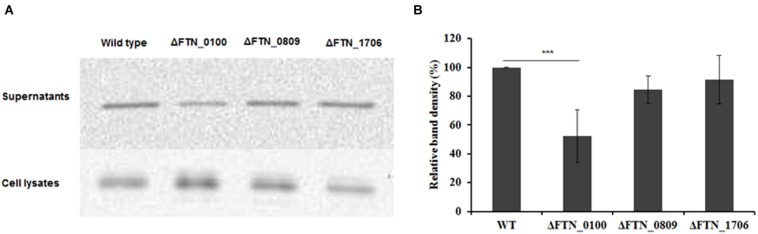
**The putative membrane protein *FTN_0100* also affects the secretion of the Type VI secretion system component, VgrG.** *FTN_0100*, *FTN_0809*, and *FTN_1706* mutants and WT strain carrying a VgrG-Flag expression plasmid were cultured in mTSB pH 7.5 in 125 ml conical flaskes to an OD_600_ of 0.3. Proteins from supernatants were collected by ultracentrifugation and TCA precipitation. Proteins from the cell lysates were obtained by sonication. Equal amounts of proteins from the supernatants and the cell lysates were used for Western blotting using an anti-Flag antibody **(A)**. Densitometry data for VgrG in Western blots from three independent experiments were evaluated **(B)**. ^∗∗∗^*P* < 0.001, one way ANOVA analysis.

VgrG, a protein encoded by the FPI, is secreted in the culture supernatant as well as inside macrophages and required for phagosomal escape of *Francisella* (Barker et al., 2009; Broms et al., 2012; Hare and Hueffer, 2014). Its pattern of secretion in macrophages mirrors that of AcpA (Barker et al., 2009; Dai et al., 2012). However, the mechanism of VgrG secretion in *Francisella* is still unclear. A *vgrG*-Flag plasmid was introduced into the FTN_0809, FTN_1706, and FTN_0100 mutants obtained from the BEI Resources transposon library and WT *F. novicida*. The VgrG levels in culture supernatants were examined by Western blot using the anti-Flag antibody. As shown in **Figure 5**, VgrG presence in the supernatant was decreased 50% in the FTN_0100 mutant but not in the other two mutants, indicating and involvement of FTN_0100 in the secretion of VgrG.

## Discussion

Upon infecting the host, *F. tularensis* primarily resides in the macrophage and enters via engagement by multiple host cell receptors, ultimately evading numerous host defense mechanisms in order to proliferate (Barker et al., 2009; Jones et al., 2012). Oxidative mechanisms are among the known host defense responses to bacterial infection. In our previous studies we demonstrated that acid phosphatases contribute to the virulence of *Francisella* likely by inhibiting the oxidative burst via dephosphorylation of the NADPH oxidase complex components (Mohapatra et al., 2007, 2013). AcpA, a major acid phosphatase in *Francisella*, is secreted in the culture supernatant *in vitro* and inside macrophages, where it is translocated from the *Francisella*-containing vacuole into cytosol (Dai et al., 2012). However, the mechanism by which AcpA is secreted by *Francisella* is unknown.

Export of various virulence factors across bacterial cell envelope, in most cases, is mediated by sophisticated translocation machineries that recognize specific sequence motifs in the proteins to be secreted. To date, the structural and molecular mechanisms of the six secretion systems (Types I–VI) of Gram-negative bacteria, the unique mycobacterial Type VII secretion system, the twin arginine translocation system (TAT), the chaperone-usher pathway and the curli secretion machinery have been elucidated (Costa et al., 2015). Comparative genomic characterization of *Francisella* spp. demonstrates the presence of components of the TAT, Type I secretion system (T1SS), Type II secretion system (T2SS), Type V secretion system (T5SS) and Type VI secretion system (T6SS) (Champion et al., 2009). However, it is unclear which systems are functional or if they function in a manner similar to those of other Gram-negative bacteria. Although the Sec-secretion system of *Francisella* contributes to biofilm formation, it is not clear if this is a result of altered protein secretion (Margolis et al., 2010). There is no evidence to support the presence of the Type III or Type IV secretion systems or their functional role in *Francisella*.

Currently, the Type IV pili and the Type VI secretion system encoded by the FPI, to best our knowledge, there are only two functional, characterized extracellular protein secretion machineries in *Francisella* (Champion et al., 2009). Type IV pili have been shown to secrete several proteins including chitinases, PepO, and BglX (Hager et al., 2006) while the secretion of FPI-encoded proteins (e.g., IglE, IglC, IglI, PdpE, PdpA, IglJ, and IglF) was shown to be mediated by the Type VI system in *Francisella* LVS, although it lacks components found in other Type VI systems (Broms et al., 2012). Inactivation of *pilB* or *pilC* abolished the ability to secrete proteins by the Type IV pili secretion apparatus (Margolis et al., 2010), suggesting the pivotal role of these proteins for its function. In this study, inactivation of *pilB* or *pilC* of did not affect AcpA secretion (**Figure 1A**), thus the Type IV pilus is not involved in the secretion of AcpA. Furthermore, deletion of the complete T6SS in *F. novicida* did not abolish AcpA secretion (**Figure 1B**). These data provide compelling evidence showing that the two functional protein secretion machineries, Type IV pili and Type VI, do not secrete AcpA. Additionally, although AcpA has been found in membrane vesicles from *Francisella*, it is still detected in the supernatant after removal of these vesicles by high speed centrifugation (Dai et al., 2012). Finally, simple cell lysis is also not responsible for supernatant AcpA because we did not detect two known cytosolic proteins (PmrA and N-terminally deleted β-lactamase) or the membrane protein FopA in the culture supernatant (**Figures 1** and **3**; Supplementary Figure S1). It is thus likely that AcpA is secreted by non-classical secretion machinery.

Since AcpA is secreted independent of the membrane vesicles, the Type IV pili, or the Type II (inferred because the Type II signal sequence at the N-terminus of AcpA was not required for its secretion; Dai et al., 2012) or the Type VI secretion system, we sought to establish a protocol for the screening of a transposon library for genes involved in AcpA secretion. The separation of the proteins in the culture supernatant from membrane vesicles, cell debris, and bacterial cells is critical for an efficient screening. In an attempt to optimize a centrifugation scheme using a 96-well plate, we found that centrifugation twice at 2095 × *g* at 4°C for 40 min efficiently remove membranes and bacteria from the culture, demonstrated by the absence of the outer membrane protein FopA in the culture supernatant (Supplementary Figure S1). We also developed an ELISA technique to quantify the amount of AcpA found in the supernatants and the whole cell fractions. Both procedures were used at various points in the screening process.

Although AcpA was found to be secreted from WT *Francisella* both *in vitro* and within macrophages (Dai et al., 2012), the AcpA levels in the culture supernatant grown in mTSB is still minimal (Supplementary Figure S1). In our attempt to maximize AcpA levels in the culture supernatant, we cultured WT *F. novicida* carrying an *acpA*-Flag expression plasmid to various stages of the growth and in various pH conditions in mTBS. To our surprise, we found that AcpA levels in the culture supernatant were dependent on the pH of medium and growth phase. The optimal growth condition in mTSB for AcpA production is pH 7.5/8.0 and bacterial OD_600_ of 0.3, which was obtained at 22 hrs post-inoculation in 96-well plate in static conditions. It is not clear how pH affects the level of AcpA and which steps of AcpA synthesis (transcription, translation, secretion or protein stability) are regulated by pH and growth phase. Nevertheless, the optimal growth conditions (pH 7.5 and OD_600_ 0.3) together with the 96-well centrifugation scheme (Supplemental Figure S1C) was used to screen *F. novicida* mutants for decreased AcpA secretion (Supplemental Figure S2).

While less than complete coverage, we screened 5,450 mutants created from WT *F. novicida* carrying an AcpA-Flag expression plasmid. Ultimately, three mutants were identified with decreased AcpA in the supernatant but not in the whole cell lysate. We did not find any mutants with increased AcpA secretion. Two mutants (*FTN_0809* and *FTN_1706*) with moderately decreased (60% in activity) AcpA secretion belong to the MFS of proteins known to be involved in fucose and drug transport, respectively. It is not known how a MFS member might affect AcpA secretion since most of the literature on MFS members refers to the transport of metabolites and drugs (Pao et al., 1998). Importantly, the *FTN_0100* mutant, encoding a hypothetical protein, has an 85% decrease in supernatant acid phosphatase enzymatic activity (**Figure 3A**), which was confirmed by Western blot (**Figures 3C,D**). It is also not clear how this protein is involved in AcpA secretion; however, it is predicted to be a membrane protein with nine alpha-helical transmembrane domains and potential protein transport activity by both the *I-TASSER Suite* (Yang et al., 2015) (Supplementary Figure S3) and *Phyre2* (Kelley et al., 2015) (data not shown). No signal sequence was detected by SignalP (Emanuelsson et al., 2007) in FTN_0100 (data not shown). *FTN_0100* mutation does not seem to affect membrane integrity since there is no difference in minimum inhibitory concentrations (MICs) in mTSB of sodium dodecyl sulfate (0.16% w/v), ethidium bromide (3.125 μg/ml), and deoxycholic acid (0.78 μg/ml) between the WT and the mutant (data not shown). The *FTN_0100* mutation also affected the secretion of the FPI protein VgrG, a predicted trimeric membrane puncturing complex located at the tip of the Type VI secretion apparatus (**Figure 5**), which was demonstrated to be secreted via the Type VI system in the LVS strain but not in *F. novicida* (Broms et al., 2012). Interestingly, genomic analysis showed that *FTN_0100* is truncated in the LVS and SCHUS4 strains, although functional activity assays of these truncated proteins has yet to be performed. Additionally, outside of the francisellae, there are no strong identity matches to FTN_0100, suggesting a potential uniquely adapted secretion mechanism in this microbe. Further characterization of FTN_0100 will shed the light on its role in the mechanism of AcpA secretion.

The study of protein secretion in the *Francisellae* is still in its infancy. This organism appears to possess few traditionally recognized, functional secretion systems, and those that are present are missing components found in other bacteria or possess serotype-specific functional differences. However, it is clear that *Francisella* acid phosphatases affect the oxidative burst and that at least one member, AcpA, is secreted both *in vitro* and into the macrophage cytosol of all tested subspecies. While a novel, genetically clustered multi-component secretion system was not identified in this study, three proteins that affect AcpA secretion were identified, which are likely to provide new insight into protein transport in this bacterium.

## Author Contributions

Conceived and designed experiments: JG, KH. Performed experiments: KH, CC, JK, JM, HA. Wrote the paper: JG, KH.

## Conflict of Interest Statement

The authors declare that the research was conducted in the absence of any commercial or financial relationships that could be construed as a potential conflict of interest.

## Footnotes

1 https://www.beiresources.org
