# Supplementary material for: Identification of Genes Required for Secretion of the Francisella Oxidative Burst-Inhibiting Acid Phosphatase AcpA
Source: Front Microbiol. 2016 Apr 28;7:605. doi: 10.3389/fmicb.2016.00605 (PMC4848305; doi:10.3389/fmicb.2016.00605)

Supplementary Material

Identification of genes required for secretion of the *Francisella* oxidative burst-inhibiting acid phosphatase AcpA

Ky Van Hoang, Carolyn G. Lee, Jacob Koopman, Jasmine Moshiri, Haley E. Adcox and John S. Gunn

Center for Microbial Interface Biology, Department of Microbial Infection and Immunity, The Ohio State University, Columbus OH 43210

Corresponding author: John S. Gunn, E-mail: gunn.43@osu.edu

Supplemental Figure 1. Optimizing centrifugation schemes for the detection of AcpA in the culture supernatant by ELISA. Wild-type F. novicida (WT) without the plasmid-borne acpA-Flag was grown in mTSB pH 7.5 in a 125ml conical flask to an OD_{600} of 0.3. The cells and cell debris were separated using a different centrifugation scheme. (D) Ultracentrifugation at 10,000 x g for 20 min followed by 150,000 x g for 135 min was shown to remove cells, cell debris, and membrane vesicles from the supernatants. The membrane protein FopA served as negative control for the supernatant fraction. Using this ultracentrifugation method as a control, three centrifugation methods were tested in 96-well plates: (A) 16,100x g for 30 min, (B) 2095x g once for 40 min, and (C) 2095 x g twice each for 40 min. A volume of 50 µl of the supernatant was used to detect AcpA and FopA by ELISA. (***P < 0.001, NS= not significant, one-way ANOVA).

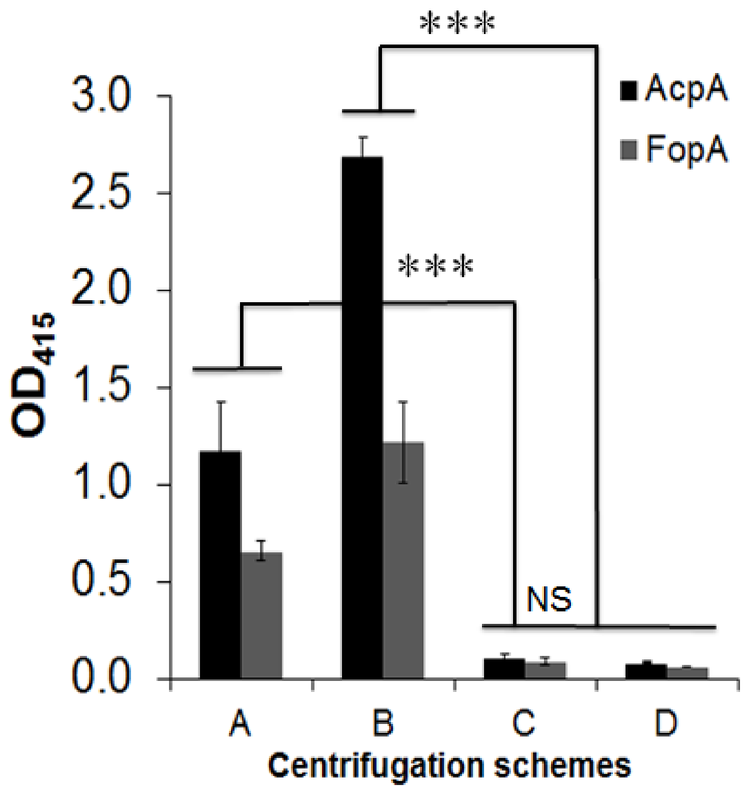

Supplemental Figure 2. Schematic diagram for the identification of genes involved in AcpA secretion. The beginning and end points of the screen are described, as well as the screening steps and putative AcpA secretion mutants identified at each stage.

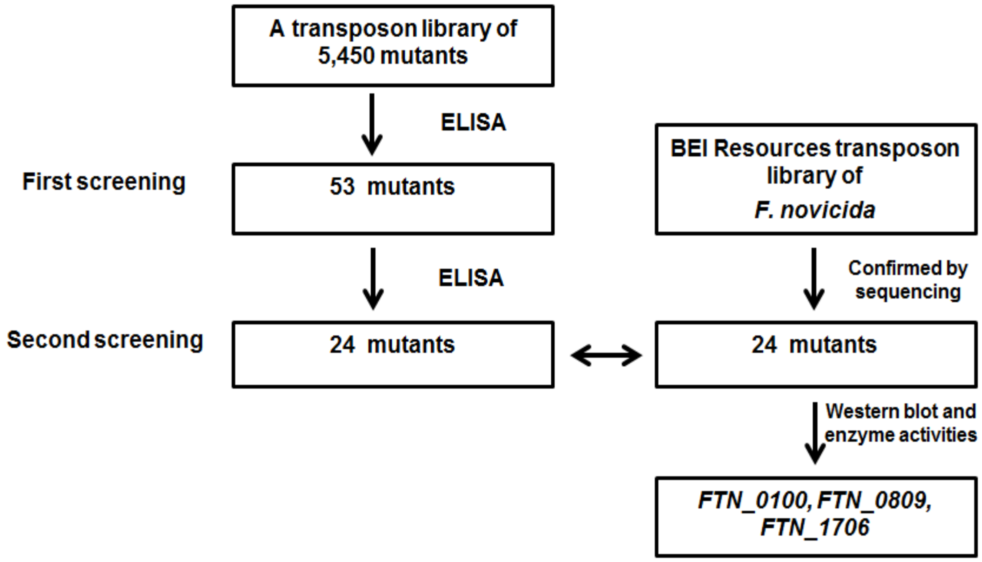

Supplemental Figure 3. Three-dimensional structure of *FTN_0100*. *FTN_0100* was predicted by the web-based software Phyr2 to be a membrane protein with nine alpha-helical transmembrane domains (data not shown). Consistent with this, the 3D structure of *FTN_0100* was predicted by the I-TASSER web-based software as possessing nine alpha-helical transmembrane domains; (A) side view; (B) Top view; and (C) bottom view.

A. **B.** **C.**

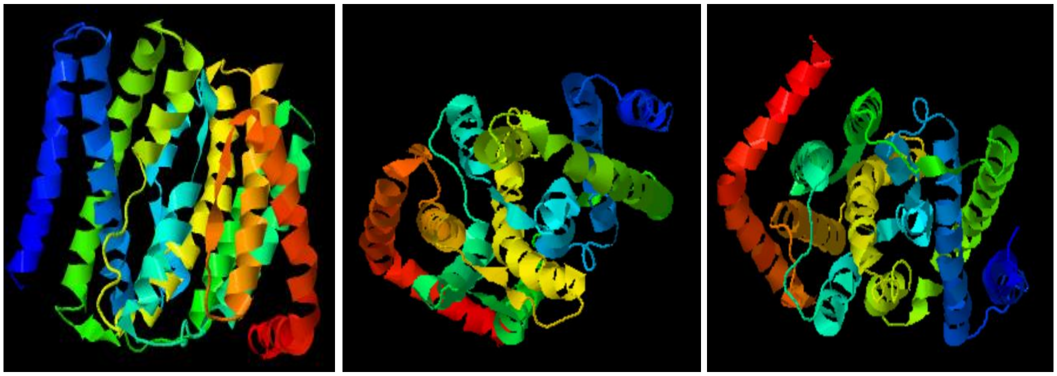

Supplement: Supplementary file 1 [file Presentation_1.PDF]
